# 

**Published:** 1897-04

**Authors:** 


### TEXAS STATE MEDICAL ASSOCIATION.

### Preliminary Announcement and Programme of the Twenty-Ninth Annual Meeting.

TO BE HELD IN THE CITY HALL, PARIS TEXAS, APRIL 27TH, 28TH 29TH AND 30TH, 1897.

COMMITTEES AND OFFICERS.

COMMITTEE OF ARRANGEMENTS—R. R. Walker, Chairman; W. M. Moore, Joseph Meyer, L. P. McCustion, J. W. Haden, B. V. Ellis, J. B. Chapman, Thos. Moody, J. M. Hooks.

RECEPTION COMMITTEE—George S. Stell, Chairman: R. R. Walker, B. F. McCustion, G. W. Bedford, W. C. Farmer, G. W. Smith, A. J. Rush, W. H. Rush.

ENTERTAINMENT COMMITTEE—L. P. McCustion, Chairman: J. W. Stephens, W. S. Baldwin.

OFFICERS.

PRESIDENT—J. C. Loggins, Ennis.
FIRST VICE-PRESIDENT—A. N. Denton, Austin.
SECOND VICE-PRESIDENT—J. S. Letcher, Dallas (deceased).
THIRD VICE-PRESIDENT—David Cerna, Galveston.
SECRETARY—H. A. West, Galveston.
TREASURER,—J. Larendon, Houston.
ORATOR—J. O. McReynolds, Dallas.

OFFICERS OF SECTIONS.

1. GENERAL MEDICINE—Lawrence Ashton, Dallas, Chairman; H. L. Tate, Lindale, Secretary.

2. Obstetrics and Diseases of Children—A. M. Douglass, Covington, Chairman; R. L. Miller, Denton, Secretary.

3. Surgery—Bacon Saunders, Fort Worth, Chairman; A. C. Scott, Temple, Secretary.

4. Medical Jurisprudence, Etc.—T. J. Bennett, Austin, Chairman; F. S. White, Terrell, Secretary.

5. Gynecology—R. R. Walker, Paris, Chairman; S. B. Kirkpatrick, Commerce, Secretary.

6. State Medicine, Etc.—I. M. Cline, Galveston, Chairman; Joseph A. Mullen, Houston, Secretary.

7. Ophthalmology, Etc.—Vard H. Hulen, Galveston, Chairman; R. F. Miller, Sherman, Secretary.

8. Dermatology, Etc,—Geo. H. Lee, Galveston, Chairman; W. M. Yater, Grandview, Secretary.

9. Microscopy, Etc.—W. R. Blailock, McGregor, Chairman; W. F. Starley, Jr., Galveston, Secretary.

### TRANSPORTATION.

The railroads generally offer a rate of one and a third fare on the certificate plan. Mr. C. E. Williams, ticket agent of the Texas & Pacific Railway, has been appointed joint agent to stamp certificates. Delegates should secure certificates from agent at point of departure which must be signed by Dr. H. A. West, and stamped as above, before they can be used to secure reduction on the return trip.

### HOTEL RATES.

Ample hotel accommodations are available. Rates as follows: Lamar, $2.00; Kimball, $1.00; Buckner, $1.25. Mrs. Tyler's, $1.25; Mrs. Jones, $1.25.

### PRELIMINARY PROGRAMME.

The session will begin Tuesday morning, April 27th, at 11:30 a. m., at the city hall. Members are requested to assemble earlier, say at 9 a. m., so as to register as far as possible before the opening. Applicants for membership will sign blank form giving postoffice address and county. Applicants not present may be admitted upon presentation of certificate signed by the President and Secretary of County or District Society. Such certificates will also be received as evidence of graduation from those who apply in person. Applicants who are not members of Societies in affiliation with the State Association will have to exhibit diploma or other satisfactory evidence of graduation.

The initiation fee has been abrogated. The annual fee is $5.00, which amount must accompany the application.

Members will receive badges when they register. All are requested to register promptly.

The titles of papers appearing in this programme having been received in time for proper order will take precedence of those offered subsequently.

ORDER OF EXERCISES.

Call to order by Chairman Committee of Arrangements, R. R. Walker, Paris.

Invocation, Rev. G. A. Farris.

Welcome by Mayor of Paris.

Welcome, representing citizens of Paris, A. P. Dohomey, Esq.

Welcome, representing Lamar County Medical Association, L. P. McCustion, Paris.

Response by the President, J. C. Loggins, Ennis.

Roll call.

Reading minutes of previous meeting.

Secretary's Annual Report, H. A. West.

Treasurer's Annual Report, J. Larendon.

President's Annual Message and Recommendations, J. C. Loggins.

The President's address and the annual oration will be delivered on Thursday evening, April 29, at 8 o'clock, after which there will be a reception tendered by the medical profession of Lamar county, this being the only social function authorized by the Committee of Arrangements.

SECTIONS.

Morning session, 9 o'clock. Afternoon, 2:30 o'clock.

GENERAL MEDICINE.

1. *Report of the Chairman. A Resume of the Advances in Medicine for the past year.*—Lawrence Ashton, Dallas.

2. *Dengue.*—H. A. West, Galveston.

3. *The Rational Medical Treatment of Anthrax.*—T. C. Osborn, Cleburne.

4. *Croupous Pneumonia.*—C. O. Matthews, Terrell.

5. *Diffuse Dermatitis Following Infiltration Anæsthesia.*—Joseph Meyer, Paris.

6. *Several Unusual Symptoms Following Cocaine Poisoning.* T. W. Conerly, San Angelo.

7. *Typhoid Fever of Texas.*—D. Dupre, Dallas.
8. *Typhoid Fever.*—W. L. Robinson, Danville, Va.
9. *Heart Disease.*—P. S. Roy, Washington, D. C.

SURGERY.

1. *Report of Chairman.*—Bacon Saunders, Fort Worth.
2. *Some Remarks Upon the Surgery of the Gall Bladder.*—Geo. W. Cale, St. Louis, Mo.
3. *Re-Section of Inferior Maxilary Bone, with Report of Two Cases.*—A. C. Scott, Temple.
4. *Traumatic Injuries of the Nerves.*—M. D. Knox, Hillsboro.
5. *Talipes Varus, with Report of a Case.*—F. E. Haynes, Abilene.
6. *Surgical Operation in the Nose by New Improved Methods.*—W. F. Cole, Waco.
7. *Surgery of the Arteries*—Brad Fowler, Brownwood.
8. *Enterollthiasis, with Report of a Case.*—J. E. Neathery, Van Alstyne.
9. *Gun Shot Wounds: Two Unique Cases.*—J. T. Benbrook, Rockwall.
10. *Abdominal Surgery.*—Thos. W. Wiley, McKinney.
11. *Carbuncle.*—A. O. Scarborough, Snyder.
12. *Operative Procedure for Bowel Obstruction, with Case.* A. N. Denton, Austin.
13. *Adenoid Vegetation in the Vault of the Pharynx, and its Treatment.*—Frank D. Boyd, Fort Worth.
14. *Perineal Section.*—Jos. Meyer, Paris.
15. *Indications for a Enucleation.*—Jno. O. McReynolds, Dallas.

GYNECOLOGY.

1. *Report of Chairman. Prophylactic Gynecology.*—R. R. Walker, Paris.
2. *Pelvic Inflammations, Etiology.*—H. K. Leake, Dallas.

   *Causes, Prevention and Diagnosis.*—F. D. Thompson, Fort Worth.

   *Pathology.*—J. T. Jelks, Hot Springs, Ark.

   *Methods of Diagnosis.*—J. M. Cable, Dallas.

   *Treatment.*—S. F. King, Sherman.

   Discussion opened by J. T. Jelks, Hot Springs, Ark.
3. *Disorders of the Mind as Relating to Pelvic Diseases.*—C. M. Rosser, Dallas.

4. *Treatment of Abortions.*—W. H. Monday, Terrell.

5. *Management of Gynecological Cases by the Country Practitioner.*—J. M. Inge, Denton.

6. *Subject Unannounced.*—Joseph Meyer, Paris.

7. *Hysterectomy, Report of Cases.*—B. F. Kingsley, San Antonio.

8. *Ectopic Gestation; Causes, Diagnosis and Treatment.*—T. A. Stoddard, Pueblo, Colorado.

9. *Puerperal Sepsis.*—C. C. Walker, Gainesville.

10. *Why Do Women Suffer?*—A. E. McMahan, Marshall.

11. *Cure of Cancer of the Uterus, with Presentation of Specimens.*—Emory Lanphear, St. Louis, Mo.

OPHTHALMOLOGY, OTOLOGY, ETC.

1. *Report of Chairman.*—Vard H. Hulen, Galveston.

2. *Optic Neuritis.*—R. H. Clilton, Dallas.

Discussion opened by R. E. Moss, San Antonio.

3. *Foreign Bodies in the Eye, and their Removal.*—E. P. Daviss, Houston.

Discussion opened by J. O. McReynolds, Dallas.

4. *The Use of the Actual Cautery in Hypopion Keratitis.*—W. R. Thompson, Fort Worth.

Discussion opened by H. L. Hilgartner, Austin.

5. *Antiseptic Technique of Cataract Operation.*—J. A. Mullen, Houston.

Discussion opened by G. P. Hall, Galveston.

6. *Scleritis and Episcleritis.*—J. O. McReynolds, Dallas.

Discussion opened by J. A. Mullen, Houston.

7. *The Oculist and the Code of Ethics.*—R. F. Miller, Sherman.

Discussion opened by R. H. Chilton, Dallas.

8. *The Toilet of the Injured Eye.*—S. J. Terrell, Dallas.

Discussion opened by E. P. Daviss, Houston.

9. *Corneal Ulcers.*—R. E. Moss, San Antonio.

Discussion opened by F. D. Boyd, Fort Worth.

10. *Hyperœsthesia Rhinitis.*—J. A. Mullen, Houston.

Discussion opened by W. R. Thompson, Fort Worth.

11. *Eye Strain as a Causative Factor in Disease of the Eyelids.*—G. P. Hall, Galveston.

Discussion opened by R. F. Miller, Sherman.

12. *Chronic Naso-Pharyngeal Catarrh.*—F. D. Boyd, Fort Worth.

Discussion opened by W. F. Cole, Waco.

*13. The Otologist's Bete Noir—Tinnitus Aurium.*—Vard H. Hulen, Galveston.

*14. Thrombosis of the Lateral Sinus.*—B. F. Church, Dallas.

The indications are favorable for a large attendance at Paris. The above list of papers guarantees an interesting and instructive session.

If the bill now pending in the legislature, regulating the practice of medicine in Texas, is enacted, the State Medical Association will be called upon to nominate certain members of the examining board.

The Committee of Arrangements have made every necessary preparation. The attendance and co-operation of every regular and reputable physician in the State is urgently solicited. County and district societies wishing affiliation with the State Association are requested to send representatives with a report of officers and membership.

H. A. WEST, Secretary.

